# Goals rather than predictions determine the sense of agency

**DOI:** 10.1016/j.isci.2025.112583

**Published:** 2025-05-05

**Authors:** Marcel R. Schreiner, Bence Neszmélyi, Katharina A. Schwarz, Wilfried Kunde

**Affiliations:** 1 Department of Psychology III, Julius-Maximilians-Universität Würzburg, 97070 Würzburg, Germany; 2 Department of Psychology, Trier University, 54290 Trier, Germany

**Keywords:** Social sciences, Research methodology social sciences

## Abstract

The sense of agency (SoA) denotes an agent’s impression of controlling environmental outcomes through acting. Most theoretical approaches assume that matching predicted and actual perceptual feedback from actions creates SoA (comparator model). We propose, however, a simpler ideomotor mechanism, which proposes SoA to emerge from matching perceptual feedback to action goals. In two experiments, participants aimed at target areas on a screen and received manipulated visual feedback. The two models predict different SoA magnitudes based on the appropriateness of the executed motor activity for achieving the goal and the intendedness of the obtained feedback. In line with the ideomotor model, but contrary to the comparator model, SoA was determined solely by the match of feedback to the goal, regardless of motor activity appropriateness. This suggests that assumptions of the comparator model should be reconsidered, specifically, that predictions do not have to be assumed to explain the emergence of SoA.

## Highlights

• The sense of agency is driven by a match between perceptual feedback and goals
• The sense of agency is not driven by a match between predicted and actual feedback
• A parsimonious ideomotor model explains the emergence of the sense of agency well
• The classical comparator model requires reconsideration

## Graphical abstract

**Figure:**
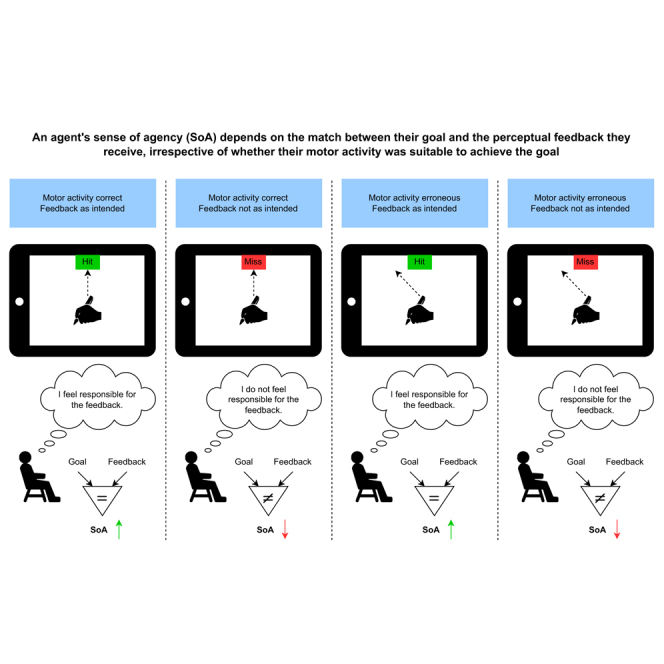

## Introduction

The sense of agency (SoA) denotes an agent’s impression to control their own actions, and through these actions events in the environment.^1^^,^^2^ This allows agents to distinguish between changes in their environment caused by themselves from those caused by other factors. SoA is involved in a number of cognitive phenomena, such as feelings of responsibility for actions,^3^ action preparation,^4^^,^^5^ and allocation of attention.^6^^,^^7^ Anomalies in the SoA are associated with severe mental illness,^8^ such as passivity symptoms in schizophrenia (i.e., beliefs that one’s thoughts and actions are controlled by external forces).^9^^,^^10^

**Figure 1:**
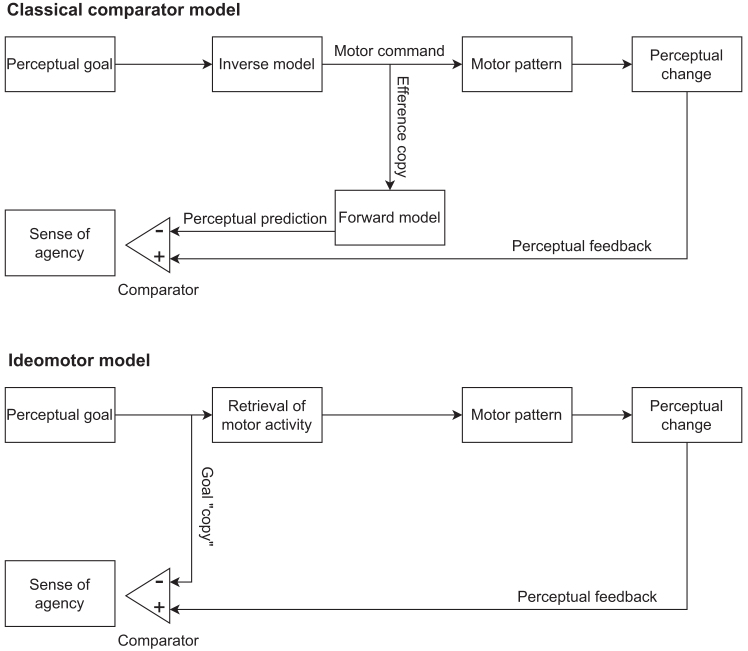
The classical comparator model and the ideomotor model for explaining the emergence of the sense of agency In the classical comparator model, the sense of agency emerges via a comparison between a perceptual prediction and perceptual feedback after action execution. The perceptual prediction is generated by a forward model, which takes an efference copy of a motor command generated by an inverse model based on an agent’s perceptual goal as input. In the ideomotor model, the sense of agency emerges via a comparison between an agent’s perceptual goal and perceptual feedback from the environment.

Early theories typically explained the emergence of the SoA by an influential comparator model^1^^,^^11^^,^^12^ derived from research in motor control.^13^^,^^14^^,^^15^ According to this model (henceforth called the classical comparator model, see Figure 1), an agent’s perceptual goal (e.g., wanting a light to be turned on) triggers an inverse model computing a command for a motor pattern required to achieve the goal (e.g., a hand movement to press a light switch). The execution of the motor pattern (i.e., the actual action) results in a perceptual change of the agent’s environment (e.g., the light being turned on). Crucially, a copy of the motor command (i.e., an efference copy) is also fed into a forward model which predicts the perceptual consequences of the preluded motor command (e.g., the light being turned on) before action execution. In the context of the classical comparator model, a prediction is therefore the output of a forward model simulating the perceptual outcome following an action based on a motor command used as an input signal. A high SoA is supposed to emerge when there is a match between the prediction and the perceptual feedback from the environment after execution of the motor pattern, whereas SoA decreases with increasing degrees of mismatch, or prediction errors (e.g., the light not being turned on after the light switch was pressed, or the light having a different color than predicted). Such a “narrow” version of the classical comparator model has been discussed broadly regarding the SoA.^1^^,^^9^^,^^16^^,^^17^^,^^18^ Updated versions of the model take a more nuanced approach as they propose that SoA emerges from the interplay of multiple processes. For example, models sometimes also include a comparison between intended and actual states,^19^^,^^20^ but this comparison is ascribed a role for motor learning, rather than a special role for the emergence of SoA in neurotypical agents, which is supposed to rest on the comparison of predicted (e.g., predicted body configurations or action outcomes) and actual states.^1^ Moreover, comparators have been assumed to operate at different levels of abstractness of action intentions, with SoA arising primarily at the level of proximal intentions (i.e., intentions associated with specific actions, e.g., to press the light switch with the right hand by extending the right arm).^21^ Yet, even these models assume SoA to emerge from a comparison between predicted and actual action outcomes. Finally, cue integration approaches have assumed that, beside internal motoric signals, external situational cues also contribute to the SoA, as evidenced, for example, by studies in which the SoA was influenced through the priming of self-attribution (presenting subliminally the word “I” or “me” before performing the action) or of the expected action effect (presenting the effect of the action before the action is performed).^22^^,^^23^ Importantly, also these extensions of the classical comparator model generally assume that its main mechanisms hold, as long as motor activity and thus outcome “prediction” is involved.

The classical comparator model can explain many phenomena related to the SoA, such as the reduction of the SoA with decreasing temporal contiguity^24^^,^^25^ or spatial incongruency between action and effect.^26^^,^^27^^,^^28^^,^^29^ However, there are observations that appear inconsistent with the classical comparator model. For instance, humans claim responsibility for typos that unbeknown to them were corrected, and accept responsibility for typos that unbeknown to them were inserted.^30^ Thus, perceived responsibility seems to depend on the perceived visual feedback and less so on the actual motor activity. Moreover, in speech production, cortical responses to perceptual auditory feedback depend on a match of that feedback to a perceptual auditory goal, rather than on a match to a perceptual prediction based on motor activity.^31^ Consequently, the premature application of the classical comparator model to phenomena beyond motor control has come under criticism.^32^ The concept of efference copies has further been questioned and it has instead been suggested that motor control rests on a comparison between intended and actual stimulation alone, rather than on a comparison between predicted and actual stimulation.^33^ In the classical comparator model, the agent’s goal appears like an input signal to the prediction machinery that shapes SoA^1^ (see Figure 1). This tends to neglect or underemphasize that the very reason to act is to reduce discrepancies between intended perception (goals) and actual perception,^34^^,^^35^ suggesting that the success or failure in reducing exactly these discrepancies between goals and perception through acting is what drives or suppresses the impression of an SoA.

Taking the fundamental goal-directedness of human motor activities into account we thus suggest that the SoA arises from a match between perceptual feedback from an action to the agent’s perceptual goal rather than from a match to the agent’s perceptual predictions. This hypothesis is inspired by recent developments of ideomotor theory.^36^ Ideomotor theory proposes that efferent activities become linked to perceptual changes which these activities produce. The same activities become retrieved if agents pursue these perceptual changes later.^37^^,^^38^^,^^39^ Thus, the model proposes that efferent activities are generated by codes of perceptual goals.^40^ We conjecture that the same codes of intended perceptual effects underlie both, action production and, through a comparison with actual feedback, also the emergence of the SoA (Figure 1). In this (more parsimonious) model, the SoA is high if the perceptual feedback matches the agent’s desired goal state and is reduced if discrepancies occur. The role of goal achievement in SoA has been investigated in numerous studies.^20^^,^^41^^,^^42^ The approaches adopted in these studies, however, generally relied on a more abstract definition of goals, as something that can be decoupled from action control processes.^20^^,^^21^ As such, in these studies, the evaluation of body-related feedback and environment-related goals were conceptualized as complementary processes. The ideomotor framework instead presupposes that goals are inherent in any action, and that they encompass both body-related and environment-related effects without a sharp distinction between their roles. Relying on this perspective, we examine the possibility that a comparator based on goals might make the presumption of the classical comparator superfluous.

To evaluate the role of the classical comparator model and the ideomotor model in the emergence of the SoA, one needs to create a rare situation in which the two models make diverging predictions. Such divergence can occur when motor activities, and the predictions of perceptual outcomes they prompt according to the comparator model, mismatch actual perceptual action feedback. There are two types of such mismatches. Either motor-based predictions suggest that the goal will be met, while actual feedback shows that it is missed, or motor-based predictions suggest that the goal will be missed while actual feedback shows that it is met. Many previous studies employed procedures involving continuous manipulated feedback on movements (e.g., a mouse cursor diverging from the actual movement trajectory).^41^^,^^42^^,^^43^^,^^44^ However, the two models do not necessarily make diverging predictions every time there is a mismatch between predicted and actual feedback. For example, while the classical comparator model would explain a reduced SoA given manipulated continuous motor feedback due to violations of motor-based predictions, the ideomotor model could also explain this phenomenon due to the violation of body-related perceptual goals. In a situation where participants achieve the intended environment-related effect despite performing an incorrect action, a possible reduction in the feeling of control could be explained by participants not achieving the body-related goals (e.g., seen and felt trajectory of a moving effector) that are also associated with the correct action. To clearly implement cases in which the two models make different predictions, we engaged participants in rather ballistic motor activities and removed perceptual feedback from movement execution that would otherwise allow the evaluation of body-related visual goals and motor adjustments on the fly. Other studies that involve environment-related effects/goals often do this to compare the influence of such factors and that of body-related effects.^41^^,^^42^^,^^45^ We were, however, not interested in this comparison. Instead, we used the manipulation of the environment-related stimuli to contrast the effects of violating predictions and intentions. We aimed to model a situation where a single motor activity is generated, and the proposed processing flows depicted in Figure 1 are run through once per individual trial.

**Figure 2:**
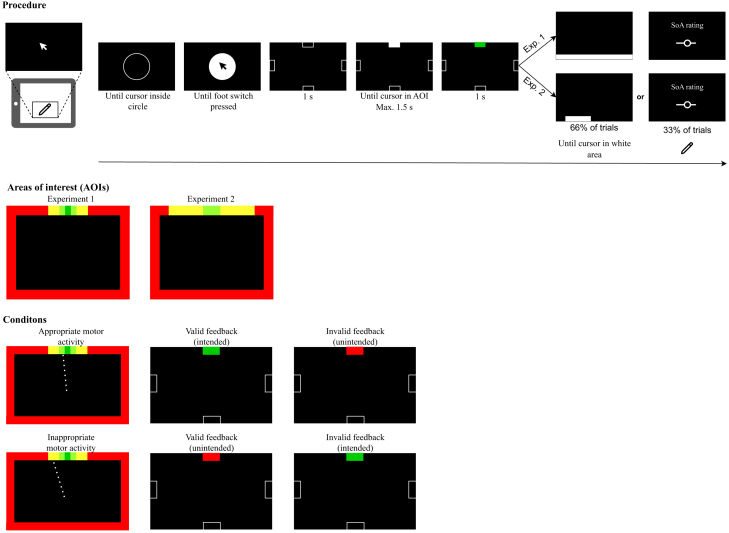
Experimental procedure, areas of interest and possible outcomes (valid or invalid feedback resulting in an intended or unintended visual outcome depending on the appropriateness of the motor activity) Each trial started with a calibration phase in which participants first had to move the pen into the outline of a circle. The circle was then filled white and the mouse cursor became visible. Participants had to press the foot switch while the cursor was inside the circle. Then, the main task started, where participants were asked to hit one of the target areas. This was followed by the secondary task and by the sense of agency (SoA) assessment in Experiment 1 and by one of these two tasks in Experiment 2.

The experimental procedure is shown in Figure 2. Sixteen participants in each experiment executed movements by moving a pen on a graphics tablet and pen movements were proportionally projected as mouse movements to the monitor. Participants were placed in a diminished feedback environment in which they could only receive the (manipulated) visual feedback besides kinesthetic feedback: for the most part of the experiment, the mouse cursor on the monitor was invisible, and the participants’ arms were covered by a cloak spanned over a wooden construction covering the graphics tablet, to mask visual action feedback other than what is presented on the screen.

Participants’ task was to move the cursor into a highlighted target area with a linear ballistic movement. They then received visual feedback depending on the area of interest (AOI) they hit and the feedback condition of this trial (see Figure 2). If they hit the target area (“hit”, green areas in Figure 2), their motor activity was appropriate, taking the relationship between hand movement and invisible cursor movement into account, and they either received correct hit feedback resulting in the intended visual outcome (the target area turned green) or incorrect miss feedback resulting in the unintended visual outcome (the target area turned red). If participants hit an AOI outside the target area, their motor activity was inappropriate and they either received correct miss feedback resulting in the unintended visual outcome (the target area turned red) or incorrect hit feedback resulting in the intended visual outcome (the target area turned green). Manipulated feedback was only given for AOIs within a certain range around the target area (“near misses”, yellow areas in Figure 2). For more remote AOIs (“far misses”, red areas in Figure 2), they always received valid miss feedback. In Experiment 2, we increased the size of the area in which responses were categorized as near misses to be able to assess the influence of larger movement errors on the SoA.

After the main task (trying to hit the target), participants conducted a secondary task in which a white target area appeared on the side of the screen opposite the target area and participants had to move the cursor into this area as soon as possible. This part of the trial always appeared in Experiment 1 but appeared in only two-thirds of the trials in Experiment 2 to separate it from the collection of SoA ratings, thus preventing the possibility of SoA ratings being influenced by the secondary task. The secondary task served the purpose of assessing the influence of the appropriateness of the motor activity and the visual feedback on subsequent movement initiation times, specifically for assessing post-error slowing.^46^ Post-error slowing refers to increased reaction times in a task following incorrect relative to correct responses and reflects increased response caution after events when the cognitive system recognizes that an inappropriate action was performed.^47^ Although we had no prior information whether the post-error-slowing effect could be obtained in the present paradigm, we included this secondary task to explore if it could serve as confirmation for the registration of inappropriate motor activity.

After some trials, participants rated their SoA by indicating how responsible they felt for the coloring of the target area. Ratings were given using a slider with possible values ranging from 0 (*no control*) to 100 (*full control*). In Experiment 1, the SoA was assessed every fifth trial, or every second occurrence of a movement categorized as an outer hit or a near miss. In Experiment 2, it was assessed after one-third of the trials (alternating with trials for assessing initiation times in the secondary task). Note that we manipulated the specific movement-related goal by varying the location of the target area, resulting in varying demands regarding the necessary motor activity to achieve the goal. While we induced the goal of hitting the target area by giving participants corresponding instructions, the intention to hit the target area was not directly manipulated. To account for the possibility that participants may sometimes have followed a deviant intention of *not* hitting the target area, we provided manipulated feedback only for the target AOIs (i.e., for hits) and for AOIs within a limited range around the target area (i.e., for responses categorized as near misses), assuming that such deviant intentions would result in movement trajectories that grossly deviate from the target area. For example, participants may intentionally execute a movement toward the opposite side of the screen. In this case, they always received valid miss feedback (which in this case matched their intention), but we did not consider the corresponding SoA ratings for the analyses. In addition, the distributions of the endpoints of the participants’ movement trajectories (see Figure S1 in the supplemental information) suggest that participants indeed tried to hit the target area, thus adhering to the instructed goal. Therefore, the results very likely reflect the case in which the participants’ intention was to hit the target area as instructed.

**Figure 3:**
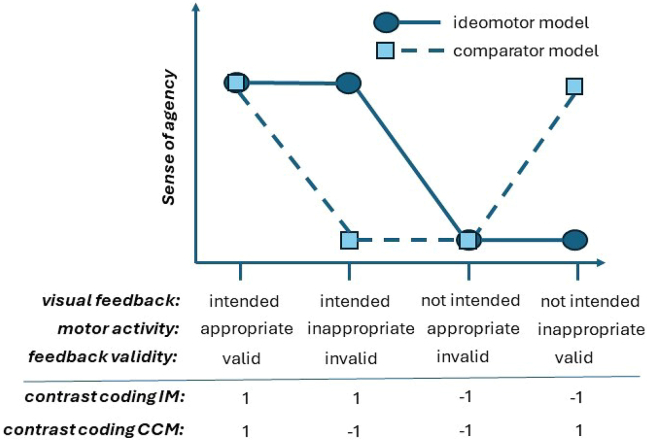
Predictions of the classical comparator model (CCM) and the ideomotor model (IM) for the magnitude of the sense of agency for the four combinations of visual feedback and motor activity The CCM predicts the sense of agency to be high if feedback is valid (visual feedback matching the motor activity) and low if feedback is invalid. The IM predicts the sense of agency to be high if visual feedback matches the agent’s intention and low if visual feedback does not match the agent’s intention, irrespective of the appropriateness of the motor activity. Additionally, the figure shows the contrast coding reflecting the model predictions used for the analyses.

The classical comparator model and the ideomotor model make different predictions regarding the magnitude of the SoA as a function of the (manipulated) feedback and the appropriateness of the executed motor activity (see Figure 3). The classical comparator model holds that the executed motor activity triggers a forward prediction of the perceptual outcome of the action. This prediction is perhaps not a hundred percent accurate, but it must be assumed to be reasonably accurate, otherwise it would be of no added value for action control and beyond. Consequently, the comparator model predicts an interaction of actual appropriateness of the motor activity and perceptual feedback. SoA should be high in cases where the feedback for the action is valid, that is, the visual feedback matches the prediction derived from the executed motor command proposed in this model (i.e., an appropriate motor activity results in intended feedback, or an inappropriate motor activity results in unintended feedback) and low otherwise. Previous research has shown that SoA declines with decreasing probability of reaching a certain perceptual feedback.^41^ Yet, the two crucial tests for the comparator model, namely that SoA should remain high if a certain perceptual feedback is not obtained *and* motor-based predictions suggest this to happen, whereas SoA should be low if that feedback is obtained *despite* motor-based predictions suggest this to not happen, has to our knowledge never been tested. The ideomotor model, however, lives without forward predictions. It assumes that there is a perceptual goal, which retrieves a certain motor activity that has become linked to the intended perceptual outcome. The goal is the best “prediction” the system can reasonably make about the outcome of a motor activity, which is the very reason for why that specific motor activity is chosen. Therefore, the ideomotor model predicts a main effect of feedback, such that the SoA should be high if the feedback matches the agent’s intention (no matter whether the motor activity was objectively appropriate or not), and low otherwise.

## Results

In two experiments, participants had to perform linear ballistic movements to hit highlighted target areas on the screen. Only at the beginning of a trial was the mouse cursor visible once participants moved it into a circle. This served the calibration of the cursor toward the screen center while allowing for variability in starting positions. In addition, participants performed a secondary task in which they had to hit an area on the side of the screen opposite the target area as quickly as possible. In Experiment 1 this area spanned the whole side of the screen, in Experiment 2 the area spanned one-third of the side of the screen, appearing in a random position. We expected that this change would result in more targeted movements, reducing the number of premature response onsets. In total, participants conducted 480 trials, preceded by 40 practice trials in which they always received valid feedback.

To compare the classic comparator model and the ideomotor model regarding their fit to the SoA data, we contrast-coded the outcomes created by crossing the factors visual feedback (intended or unintended) and motor activity (appropriate or inappropriate) to reflect the respective model prediction: the coding reflected the intended vs. unintended visual feedback contrast for the ideomotor model and the valid vs. invalid feedback contrast for the classical comparator model (see Figure 3). For each model, we then fit a Bayesian mixed linear model^48^^,^^49^ with SoA ratings as the dependent variable, the contrast-coded outcome variable as a fixed effect, and random person intercepts. To assess which model is better suited to explain the data, we conducted Bayesian model comparisons by computing Bayes factors (BF) in favor of the ideomotor model.

### Experiment 1

**Figure 4:**
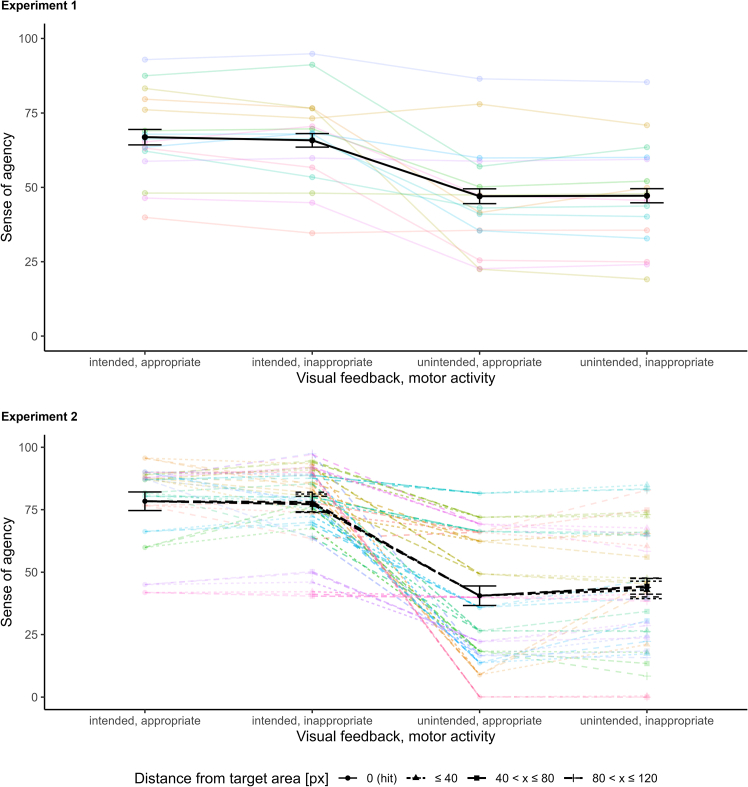
Mean sense of agency ratings for the four combinations of visual feedback (intended or unintended) and motor activity (appropriate or inappropriate) in experiments 1 and 2 For Experiment 2, ratings are shown separately for the first three “near miss” categories. In the case of appropriate motor activity there is only one category (hits) and hits were the same in all models. Colored shapes represent person-level means (each color represents one participant) and black shapes represent grand means. Error bars represent ± within-subjects SEM.

In Experiment 1, we distinguished between “central hits” if participants hit the central part of the target area (central 20 px of the target area, dark green area in Figure 2) and “outer hits” if participants hit a more remote part of the target area (outer 20 px on each side of the target area, light green areas in Figure 2). Manipulated feedback was only given for movements categorized as an outer hit, and for movements categorized as a near miss, for an area spanning 40 px next to each side of the target area. Thus, only these trials were considered for the analysis. The model comparison yielded a BF of 3.56 × 10^116^ (classical comparator model: *M* = −1.87, 95% HDI [−2.79, −0.97], ideomotor model: *M* = 10.41, 95% HDI [9.57, 11.21]), thus yielding extremely strong evidence in favor of the ideomotor model. This also matches with the mean pattern of SoA ratings displayed in Figure 4, which closely corresponds to the pattern predicted by the ideomotor model.

Despite the strong support for the ideomotor model one may argue that the efference copy is not accurate enough to track small deviations in movement trajectories that differentiated between hits and near misses in the present study. According to his objection we could expect that a pattern conforming to predictions of the comparator model would emerge when errors are more obvious to the participants. This is why we increased the size of the area for which participants could receive manipulated feedback in Experiment 2. Moreover, we implemented the SoA ratings directly after the feedback screen in Experiment 2 to account for any possible decay of forward predictions which might have prevented an influence of such predictions on SoA judgments.

### Experiment 2

In Experiment 2, manipulated feedback was given for movements categorized as a hit, for the whole target area (60 px), and for movements categorized as a near miss, for an area spanning 400 px next to each side of the target area. However, for the main analysis we only considered three near miss categories with a distance up to 40 px, between 40 and 80 px, and between 80 and 120 px from the target area. This covered most of the naturally occurring variation in movement trajectories, as, on average, only 20% of responses fell into a more distant category. When considering hits and near misses up to 40 px from the target area (cf. Experiment 1), the model comparison yielded a BF of 5.70 × 10^125^ (classical comparator model: *M* = 0.62, 95% HDI [−1.15, 2.33], ideomotor model: *M* = 19.25, 95% HDI [17.88, 20.65]), again yielding extremely strong evidence in favor of the ideomotor model. This was also the case when considering hits and near misses between 40 and 80 px from the target area (BF = 2.38 × 10^98^, classical comparator model: *M* = 4.64, 95% HDI [2.89, 6.45], ideomotor model: *M* = 17.95, 95% HDI [16.55, 19.39]), and when considering hits and near misses between 80 and 120 px from the target area (BF = 3.58 × 10^92^, classical comparator model: *M* = 6.85, 95% HDI [4.90, 8.72], ideomotor model: *M* = 19.21, 95% HDI [17.71, 20.71]). This again also matches with the mean patterns of SoA ratings displayed in Figure 4, which closely correspond to the pattern predicted by the ideomotor model for all categories, with no discernible effect of the deviation of the response from the target area. We further confirmed this in a post-hoc analysis in which we tested whether the (continuous) distance of responses resulting from an inappropriate motor activity (i.e., near misses) from the center of the target (see also Figure S1 in the supplemental information) area had any incremental effect to the visual feedback on the SoA ratings by comparing a model with the effect-coded visual feedback variable with a model with an additional distance variable as a covariate. The model comparison yielded a BF of 0.07, indicating strong evidence against an effect of distance.

### Supplementary analysis of movement initiation times

As a supplementary analysis, we tested whether inappropriate motor activities or visual error feedback increased movement initiation times in the secondary task. This was done to probe post-error slowing,^46^^,^^47^ which may suggest a detection of an inadequate movement (be that either based on inappropriate motor activity or on perceptual error feedback) by the participants’ neuro-cognitive system, consequently prompting an orienting response. In neither experiment was there such slowing, neither after inappropriate movements (Experiment 1: BF = 0.06, *M* = 2.08, 95% CI [−2.89, 6.89], Experiment 2: BF = 45.25, *M* = 10.68, 95% CI [5.17, 16.20]) nor after visual error feedback (Experiment 1: BF = 0.05, *M* = −1.54, 95% CI [−6.43, 3.37], Experiment 2: BF = 0.92, *M* = 6.69, 95% CI [1.13, 12.17]). Note that negative values of the mean of the posterior distribution indicate post-error slowing and positive values indicate post-error speeding. Conceivably, the prevalence of movement errors (Experiment 1: 69%, Experiment 2: 76%) and visual error feedback (Experiment 1: 63%, Experiment 2: 50%) was too high to prompt such a response. In contrast with the response caution theory, an orienting account suggests that post-error slowing reflects a response of the neuro-cognitive system to rare events.^50^ Thus, as movement errors and visual error feedback were rather frequent, even an opposite effect (i.e., post-error speeding) may be expected. Indeed, in Experiment 2, there was, overall, evidence for post-error speeding, which is in line with such an orienting account.^50^

## Discussion

In two preregistered experiments we compared the influential classical comparator model^1^^,^^11^^,^^12^ with a model based on ideomotor theory^36^ and previously proposed models^37^^,^^38^^,^^39^ regarding their suitability in explaining the emergence of the SoA. Specifically, we question the necessity of the assumption of a forward model that uses an efference copy of a motor command to generate predictions that are compared with perceptual feedback. Instead, we show that an ideomotor model based on the direct comparison of the perceptual goal to the perceptual feedback provides a better fit for the observed data.

In both experiments, we found very strong evidence in favor of the ideomotor model over the classical comparator model. Participants’ SoA was high if the (manipulated) perceptual feedback matched their instructed goal and was low if it did not, with little to no effect of the actual appropriateness of the executed motor activity. Thus, the more parsimonious ideomotor model could better explain the emergence of the SoA in the current study.

Our results extend previous research on the question whether SoA is driven by a comparison between actual and intended outcomes (ideomotor model) or actual and predicted outcomes (classical comparator model). Sato and Yasuda^51^ (Experiment 3) had participants execute one of two keypresses, which were each assigned a tone of certain pitch. The keypresses sometimes produced the predicted tone (a so-called action-congruent tone) whereas they sometimes did not (i.e., an action-incongruent tone). The action-incongruent tone of one action was the action-congruent tone of the respective other action. The crucial question was how action errors affect SoA. Action errors were construed as actions where the actual outcomes did not match the intended outcomes. Action errors came with a reduced SoA, which suggests a role of the comparison between intended and actual outcome in line with our conclusions. Yet, even when participants produced incorrect motor activity, SoA was reduced when that motor activity produced an action-incongruent tone that it typically would not produce, suggesting also a role of the comparison between actual outcomes and motor-based predictions. However, the authors excluded in their study errors participants did not notice. We would argue that exactly these “unnoticed errors” are particularly interesting. In such cases, an inappropriate action produced an “incongruent” tone, namely the intended tone of the correct, but not chosen action alternative. We would argue that these errors might go unnoticed because here intended and actual outcome did match. Moreover, we would suggest that SoA is higher when the erroneous motor activity produced an action-incongruent but actually intended effect, as compared to erroneous actions that produced an action-congruent but unintended effect. This is exactly what we observed here.

The results obtained in the present study are similar to findings reported in previous studies about the relationship between SoA and performance.^20^^,^^44^ It has been shown that an increase in performance results in an increase of SoA as well, even if the performance improvement is due to computer assistance and not caused by participants having better control over the outcome of their actions. In these experiments, participants are required to direct a dot on the screen toward a target area by controlling its movement with directional keys. In the computer assisted condition, participants’ keypresses only result in the movement of the dot if they move the object in the direction of the target area. Movement of the dot is blocked, however, if participants press a key that would guide the object in the wrong direction. That is, participants’ actual control over the movement of the object, and the possible contributions of a classical comparator are reduced in this condition, participants’ judgments, however, indicate an increased SoA. In these dot-control studies, goals refer to the overarching objective that can be achieved with a sequence of separate actions. Thus, the authors conclude that the influence of these overarching goals is compatible with a classical comparator that can exert its influence on the level of individual actions.^20^ Our study extends these findings by showing that by regarding action effects as goals, the influence of goal evaluation on SoA also applies to the level of individual actions. Consequently, from an ideomotor perspective, the evaluation of goal achievement (i.e., intention vs. actual outcome matching) does not only complement a classical comparator but it can make the role of the latter’s mechanisms in establishing the SoA obsolete.

Therefore, also considering findings from other studies that are inconsistent with the classical comparator model,^30^^,^^31^ we suggest to reconsider the role of the classical comparator model in explaining the SoA. Our results indicate that in the rare case where the classical comparator and the ideomotor models make diverging predictions, the ideomotor model fits the empirical data better. Thus, a comparator mechanism based on the ideomotor framework might provide a more substantial contribution to explanations of the SoA than the one based on the comparison of predicted and actual feedback. Note that our results do not necessarily imply that the ideomotor model is an encompassing framework for explaining the emergence of the SoA, and additional (e.g., external) influences may be at play. However, compared to the classical comparator model and extensions of the same, it can explain empirical findings better while being more parsimonious. In the following, however, we discuss three arguments that may be put forward in defense of the classical comparator model in light of the current findings.

### Arguments for the classical comparator model

Argument 1: the predictions of the forward model are not precise enough to register deviations in the motor activity that rendered it inappropriate in the experimental setting.

While, to our knowledge, the preciseness of the prediction of the forward model in the classical comparator model has not yet been unambiguously scrutinized, we believe this argument to be problematic, as this would pose an auxiliary assumption that would render the classical comparator model essentially unfalsifiable. Moreover, it is also unlikely given our results. Particularly, in Experiment 2 we investigated larger deviations in motor responses, covering much of the naturally occurring variability in movement trajectories, and consistently found strong evidence in favor of the ideomotor model. We also investigated whether the deviation of the participants’ movement trajectories from the target area had an incremental effect to the effect of visual feedback and the appropriateness of the motor activity, which would be expected if the forward model produces noisy predictions that may not register smaller deviations. However, this was not the case. If the forward model produces predictions that are so noisy to not flag motor commands as inappropriate even if there is a clear deviation from the target area, these predictions seem quite inadequate to reliably inform control experience. One might question why we did not include trials where we manipulate the trajectory of movements in a way that errors would become even more obvious (e.g., perturbed actions hitting a different side of the tablet and still eliciting a visual effect that signals correct response). We did so because we wanted to create a situation in which the feedback regarding the success of an executed motor command is limited as much as possible to just one visual feedback signal, namely the color change of the target area. With very large perturbations, other feedback signals, specifically proprioceptive feedback, come into play, and are likely used to evaluate executed motor activities (e.g., “I wanted to hit the upper target area, but it felt like moving leftwards”). However, the question whether primary action goals (i.e., achieving the intended visual change) could still dominate SoA judgments in cases when perceptual goals in other perceptual modalities (co)exist could be an interesting topic for future research.

Argument 2: the results did not match the predictions of the classical comparator model because participants did not receive visual information regarding their continuous movement.

In our experiments, participants were placed in a very limited feedback environment in which they did not see the mouse cursor they moved during the relevant part of the trial, and their hands and arms were covered. It is indeed possible that results may have differed if the mouse cursor had been visible, as participants could have used this continuous visual feedback to infer the appropriateness of their motor activity or update their movement on the fly. However, with additional visual feedback the distinction between the predictions of the classical comparator and the ideomotor model would have been less clear: factors like lower-order visual goals^43^ (e.g., participants’ expectation that the final visual feedback is accompanied by the visual image of the cursor hitting the target area) or a retrospective comparison of movement endpoint and target area would also allow for the ideomotor model to explain patterns that are predicted by the classical comparator model. We believe the decision to use a setting with restricted feedback does not compromise our results since the classical comparator model should also apply to such scenarios and restricting its use to situations in which continuous visual feedback regarding one’s actions is available would substantially limit the generalizability of the model. In fact, the classical comparator model has been applied to situations similar to our experiments, involving discrete actions, for example, key press actions that produce tones.^52^ This is not conceptually different from the situation in our experiments, in which participants performed linear ballistic movements upon which they received perceptual feedback. Conceptually, the ideomotor framework does not distinguish between body-related and environment-related goals. Thus, we assume that the results would not change substantially, if agents could represent their actions in terms of their body-related effects. The reason for emphasizing the environment-related goals in the current study was that the inclusion of trials where the final effect matches the goals of the agent despite the erroneous action execution was of central importance to the design. While we can make relatively strong assumptions about environment-related goals (i.e., participants likely want to elicit the visual effect associated with successful performance), participants’ intentions regarding body-related effects (i.e., which exact path they planned) are less accessible.

Argument 3: given that participants were sometimes visually fed back an inappropriate motor activity as being appropriate, they learned novel motor-visual links on which they based their SoA ratings.

If participants indeed learned such transformations, then the variability of responses should be larger in Experiment 2 than in Experiment 1, as the area for which participants were fed back an inappropriate motor activity as being appropriate (i.e., intended feedback) was larger in Experiment 2. To test whether this was the case, we computed the variance of responses for each participant in each experiment, separately for the possible positions of the target area. We then tested whether the variability in responses differed between experiments by fitting Bayesian mixed linear models and computing a BF in favor of an effect of the experiment, while controlling for the position of the target area. This post-hoc analysis yielded a BF of 0.38, indicating evidence (albeit non-conclusive) against an effect of the experiment. Therefore, the variability of responses did not differ between experiments, which contradict the assumption that participants learned transformations on which they based their SoA ratings.

### Other approaches to the sense of agency

Other approaches to the SoA beyond the classical comparator model as discussed in the current research exist. Here, we briefly address three: the motor control system by Frith et al.,^19^ cue integration approaches,^22^^,^^23^ and a self-serving bias account.^53^^,^^54^

First, the motor control system by Frith et al.^19^ not only includes a forward model that generates predictions and compares them with perceptual feedback (as in the classical comparator model), but also an additional comparator that compares an agent’s desired state to perceptual feedback. Applied to the SoA, this is essentially a combination of the classical comparator model and the proposed ideomotor model. One can question, however, if the classical comparator component of the system is really necessary for explaining the emergence of the SoA. Our results suggest that it is not, and thus Occam’s razor demands to use a more parsimonious version of the model, which then essentially reduces to the proposed ideomotor model.

Second, cue integration approaches to the SoA^22^^,^^23^ assume that different types of agency cues become integrated and contribute to the SoA. These can be internal, motor-based cues, which essentially are the result of the classical comparator model, but also external, situational cues, such as priming movement-related thoughts. Crucially, cue integration approaches suggest that agents flexibly exploit cues based on their availability and reliability. For example, agents may rely more strongly on external cues as internal cues become less reliable or even absent. Thus, cue integration approaches still assume the classical comparator model to be valid but further assume that it can be downregulated under certain conditions. We do not question the contribution of external cues to agency in shaping the SoA. However, our results question whether internal cues are really the result of motor-based predictions of perceptual action consequences. Rather, our results suggest that internal cues are the result of the comparison between an agent’s perceptual goal and the perceptual feedback following action execution in the proposed ideomotor model rather than the comparison between predicted and observed perceptual feedback in the classical comparator model. This reconceptualization of internal cues would again result in a more parsimonious model. Nevertheless, one could still argue that, in cases in which the data do not fit the predictions of the classical comparator model, this is due to this component having been downregulated to zero in the respective testing situation. As is the case with the assumption of imprecise forward model predictions discussed previously, this would make the model very hard to falsify. Proponents of the classical comparator model (or its integration into broader approaches) should therefore specify what would be a critical test of the classical comparator model for which this argument cannot be applied.

Finally, an agents’ SoA may be shaped by a self-serving bias in the sense that participants tend to self-attribute positive outcomes and reject negative outcomes.^53^^,^^54^ In our experiments, such a self-serving bias would lead to similar predictions as the proposed ideomotor model, as the possible outcomes (hitting or missing a target) could be considered positive or negative, and negative outcomes were likely unintended. The current research therefore cannot distinguish between the ideomotor model and a self-serving bias account. This would require crossing outcome valence and outcome intendedness, which might be an interesting prospect for future research.

To summarize, the results of two experiments strongly suggest that the emergence of the SoA can be better explained by a more parsimonious ideomotor model than the classical comparator model,^1^^,^^11^^,^^12^ while also challenging major arguments that may be put forward in defense of the classical comparator model. Therefore, we argue that the role of the comparator model in the emergence of the SoA should be reconsidered, and its replacement with more goal-based models, such as the proposed ideomotor model, might be warranted, except if it can be convincingly demonstrated that forward model predictions substantially contribute to the SoA beyond comparisons of perceptual goals and feedback.

### Limitations of the study

A limitation of our experiments is that we do not have an indication of whether participants explicitly noticed whether they committed an error (except for the movement initiation times in the secondary task). While this does not invalidate the results, an even stronger case could be made if, for example, participants reported a high SoA after committing and consciously perceiving an error when invalid but goal-consistent feedback is given. This might be an interesting prospect for future research.

We further did not separate the effect of intentions and expectations. For example, while participants intended to hit the target area, they may have had varying expectations of success given their motor skills. It has been suggested that such expectations of (or better: prior beliefs about) the controllability of the environment tune the sensitivity of the SoA to actual control.^55^ However, a close correspondence between the SoA and the perception of the predictability of action outcomes has been observed, with the SoA being high if the outcome was highly predictable and low if predictability was low.^56^ Given that the *a priori* probability of receiving valid or invalid feedback was constant in our experiments, we consider possible effects of expectations to be unlikely to substantially influence the results, as they can be expected to be fairly constant within participants. Nevertheless, assessing participants’ confidence in their degree of motor accuracy would be a valuable addition in future research.

Another limitation is that we assessed participants’ SoA through explicit ratings. These ratings are subjective and may be influenced by cognitive and response biases. The SoA can also be assessed implicitly, using a measure of intentional binding,^52^^,^^57^ and explicit and implicit measures of the SoA sometimes converge.^24^^,^^58^^,^^59^ However, explicit and implicit measures of the SoA are often not particularly related,^60^^,^^61^^,^^62^ and the validity of intentional binding for measuring the SoA has recently been questioned.^63^^,^^64^ We thus opted for an explicit measure of the SoA. Nevertheless, it would be interesting to test whether results looked similar when using an implicit measure of the SoA, such as intentional binding or sensory attenuation.^65^

## Resource availability

### Lead contact

Requests for further information and resources should be directed to and will be fulfilled by the lead contact author Marcel R. Schreiner (marcel.schreiner@uni-wuerzburg.de).

### Materials availability

All study materials are available at the Open Science Framework (https://doi.org/10.17605/OSF.IO/4K25B).

### Data and code availability

• Data: All raw data (individual datasets) are available at the Open Science Framework (https://doi.org/10.17605/OSF.IO/4K25B).
• Code: All original code, including analysis files and experiment code, is available at the Open Science Framework (https://doi.org/10.17605/OSF.IO/4K25B).

## Acknowledgments

We thank Colin Schwinum, Theresa Jacob, and Johannes Bernau for collecting data for the experiments, and Luca Germann for assisting in the curation of research materials. No funding was received for this research.

## Author contributions

M.R.S.: conceptualization, methodology, formal analysis, software, data curation, visualization, writing – original draft, writing – review and editing, project administration; B.N.: conceptualization, methodology, validation, visualization, writing – review and editing; K.A.S.: conceptualization, methodology, validation, writing – review and editing; W.K.: conceptualization, methodology, validation, visualization, writing – review and editing, project administration.

## Declaration of interests

The authors declare no competing interests.

## STAR★Methods

### Key resources table

**Table:** 

REAGENT or RESOURCE	SOURCE	IDENTIFIER
**Deposited data**		

Analyzed data	This paper	
Raw data		https://doi.org/10.17605/OSF.IO/TJM6Q

**Software and algorithms**		

Analysis files		https://doi.org/10.17605/OSF.IO/TJM6Q
Code of the experiments		https://doi.org/10.17605/OSF.IO/TJM6Q
OpenSesame version 3.3.5	Mathôt et al.^65^	https://osdoc.cogsci.nl/
Mousetrap plugin for OpenSesame version 2.1.0	Kieslich and Henninger^66^	https://github.com/PascalKieslich/mousetrap-os
R version 4.3.1	R Core Team^67^	https://www.r-project.org/
BayesFactor R package version 0.9.12-4.6	Morey and Rouder^68^	https://cran.r-project.org/package=BayesFactor

**Other**		

Intuos 4 XL	Wacom Co., Ltd.	https://www.wacom.com

### Experimental model and study participant details

Participants were native German speakers with normal or corrected-to-normal vision that were recruited from the participant pool of the University of Würzburg and received a compensation of 18€ for the approximately 90-minute experiments. In both experiments, we intended to employ a sequential testing approach, collecting a minimum of 16 usable datasets up to a maximum of 36 usable datasets and ceasing data collection when the Bayes factor in favor of the ideomotor model exceeded 10 (indicating strong evidence for the ideomotor model) or was below 0.1 (indicating strong evidence for the classical comparator model). For Experiment 2, this criterion was applied for three separate analyses for responses classified as near misses falling within 40 px, between 40 and 80 px, and between 80 and 120 px from the target area. In both experiments, there was already very strong evidence in favor of the ideomotor model after the minimum sample size of 16 usable datasets (Experiment 1: *M*_*Age*_ = 27 years, *SD*_*Age*_: 9.66 years, 14 women [87.5%], 2 men [12.5%] 13 right handed [81.3%], 3 left handed [18.8%] ; Experiment 2: *M*_*Age*_ = 29.38 years, *SD*_*Age*_: 8.05 years, 12 women [75%], 4 men [25%], all right handed). To reach the final sample of 16 participants, in Experiment 1, 18 participants completed the experiment: One participant was excluded due to an insufficient number of available SoA ratings (less than five ratings for outer hits or near misses in either feedback condition). Another participant was excluded because they responded too slow in more than 5% of trials. In Experiment 2, 23 participants completed the experiment: Six participants were excluded due to an insufficient number of available SoA ratings (less than five ratings for hits or for the three near miss categories closest to the target area in either feedback condition) and another participant was excluded because they responded too slow in more than 5% of trials. No ethics approval was required according to the ethical guidelines of the German Society for Psychology (DGPs) and regulations of the German Research council (DFG), as the research had no foreseeable negative impact on participants, no patients were tested, no electric or magnetic stimulation was used, participants provided informed consent, and data were collected anonymously. All individual participants included in the study provided informed consent for their participation and publication of their data. Due to the small number of male participants in the samples and because we did not expect gender to modulate the basic cognitive processes studied here, no sex- or gender-based analyses were conducted.

### Method details

#### Apparatus

Participants sat in front of a monitor with a resolution of 1920 × 1080 px and a refresh rate of 60 Hz, with a viewing distance of approximately 70 cm and a graphics tablet (Intuos 4 XL, Wacom Co., Ltd.) in front of them. Only a part of the graphics tablet was active, allowing for reasonably easy and comfortable movements from the center of this active part towards its borders and preventing the frame of the tablet from interfering with participants’ movements. The active part spanned an area of 24.5 × 15.4 cm. Participants could move a mouse cursor on the screen by moving an Intuos Ink Pen on the active part of the graphics tablet. These movements were proportionally projected as mouse movements onto the monitor. The tablet was covered by a wooden construction under which participants could reach and move freely. In addition, participants were covered with a cloak that was attached to their neck and the wooden construction, such that they could not see their hand or arm to mask visual action feedback other than what is presented on the screen. Thus, participants only received visual feedback on their actions on the monitor. Further, a foot switch was placed under the table. Pressing this switch was translated as a keypress. A keyboard and a computer mouse were placed on a slidable platform under the table, which participants could use to make inputs at the beginning and end of the experiments. The experiments were implemented using OpenSesame 3.3.5^66^ and the mousetrap plugin 2.1.0.^69^

#### Design

Both experiments employed a one-factorial (feedback condition: valid vs. invalid) within-subjects design. However, this manipulation only took effect for responses categorized as hits (for Experiment 1 only for outer hits) or near misses.

#### Procedure

At the beginning of each experiment, participants provided informed consent and demographic information (gender, age, and handedness). The experimental procedure is shown in Figure 2. Each trial started with the white outline of a circle on a black background. Participants were instructed to move the pen into the circle. The mouse cursor was invisible. When the mouse cursor entered the circle, the circle was filled white, and the mouse cursor became visible. Participants were instructed to press the foot switch while the mouse cursor was inside the circle. This part of the trial served to calibrate the starting position of the mouse towards the screen center while allowing for variability in starting positions.

Next, the mouse cursor became invisible again and participants were presented with a screen with four outlines of rectangles at the top, right, bottom, and left border of the screen, spanning 60 px of and being centrally placed on the screen borders (reaching 30 px into the screen). These indicated the possible target areas. After 1 s, one of the rectangles was filled white, indicating the target area for this trial. The position of the target area (top, right, bottom, or left) was counterbalanced across trials within the feedback conditions. Participants were instructed to perform as fast as possible a linear ballistic movement that passes through the target area. If participants took more than 1.5 s to reach any screen border, they received feedback of being too slow for 2 s and the trial was categorized as a timeout. Otherwise, participants received visual feedback for 1 s. The target area could either turn green, indicating a hit, or turn red, indicating a miss. Feedback was given based on the feedback condition and the area of interest (AOI) on the screen border the participant hit (see Figure 2).

In Experiment 1, if participants hit the central part of the target area (spanning 20 px, dark green area in Figure 2), the response was categorized as a central hit (appropriate motor activity) and they always received valid hit feedback (i.e., the target area turned green). If they hit one of the outer thirds of the target area (spanning 20 px on either side of the central area, light green areas in Figure 2), responses were categorized as outer hits (appropriate motor activity), and they received feedback depending on the feedback condition. In the valid feedback condition, they received valid hit feedback (i.e., the target area turned green, intended feedback). In the invalid feedback condition, they received invalid miss feedback (i.e., the target area turned red, unintended feedback). In Experiment 2, the distinction between central and outer hits was abandoned and participants could always receive manipulated feedback for a hit (light green areas in Figure 2). In Experiment 1, if participants missed the target area but hit an AOI next to the target area (spanning an area of 40 px on either side, yellow areas in Figure 2), the response was categorized as a near miss (inappropriate motor activity), and participants again received feedback depending on the feedback condition. In the valid feedback condition, they received valid miss feedback (i.e., the target area turned red, unintended feedback). In the invalid feedback condition, they received invalid hit feedback (i.e., the target area turned green, intended feedback). In Experiment 2, the area for which responses were categorized as near misses was extended. Instead of one AOI, there were ten AOIs spanning an area of 400 px in total (each AOI spanned an area of 40 px). Thus, different near miss categories were distinguished based on the distance of the hit AOI to the target area. In both experiments, if participants hit any other part of the screen border (red areas in Figure 2), the response was categorized as a far miss (inappropriate motor activity), and participants always received valid miss feedback (i.e., the target area turned red, unintended feedback). The *a priori* probability of receiving valid or invalid feedback across trials was 50%, also within the four possible positions of the target area.

In Experiment 1, after having received feedback and only in trials not categorized as timeouts, participants conducted a secondary task. The side of the screen opposite the target area turned white, and participants were instructed to move the pen into the highlighted area as fast as possible. This served the purpose of assessing whether initiation times in the secondary task are influenced by the appropriateness of the motor activity and the visual feedback in the previous task. In Experiment 2, the secondary task only occurred in two thirds of the trials and the highlighted area spanned only one third of the available border area (i.e., 640 px when appearing at the top or bottom and 400 px when appearing at the left or right) and appeared in a random position at the screen border. This was done to reduce the number of trials with premature response onset and to require participants to aim for hitting the highlighted area. In other words, more accurate movements were required by the participants to hit the highlighted area. This may increase post-error slowing, which has been suggested to be more pronounced in settings that emphasize accuracy,^67^ which engage more cognitive control and error monitoring. Participants’ SoA was assessed by asking them “How much do you feel responsible for the coloring of the target area?” (i.e., the feedback) after some trials. Participants could enter their response using a slider ranging from 0 (*no control*) to 100 (*full control*). In Experiment 1, SoA was assessed every fifth trial, or every second occurrence of a response categorized as an outer hit or a near miss (but never after a timeout). Participants could use the pen to move the slider and log their response by pressing the foot switch. In Experiment 2, participants’ SoA was only assessed in trials where the secondary task was not performed. Thus, in Experiment 2, we separated these two components of a trial to prevent SoA ratings from being influenced by the secondary task. The main part of the experiments consisted of 480 trials. The trial order was randomized. After 25%, 50%, and 75% of trials there was a short break of 30 s.

Before the main part of the experiments, participants received detailed instructions and were guided through the different components of a trial step by step. They also conducted 40 practice trials in which they always received valid feedback. After the main part of the experiments, participants could indicate whether there were any reasons why their data should not be used for the analyses and give general comments regarding the study. To do this, they could remove the cloak and slide the keyboard and computer mouse from under the table or ask the experimenter to do so. Finally, participants were thanked and debriefed.

The size of the target areas and AOIs was assessed in a pilot study (*N* = 3), which showed that the chosen sizes yielded a sufficient distribution of response categories, with a sufficient availability of SoA ratings within the categories. Note that we measured the SoA using explicit ratings.

During action execution, we eliminated visual feedback on the movement trajectory. This limited the possibility that participants would adjust their actions on the fly to reestablish an alignment of either predictions or goals. Furthermore, if participants had received visual feedback on the movement trajectory it would have been evident when feedback was manipulated on a trial (due to the mismatch between the target location and the end position of the hand or the visual image of the movement trajectory). In this case, if participants had accomplished the intended visual goal, arguably they would have still violated the goal formulated in the instructions (i.e., eliciting a visual stimulus by hitting the target area). Thus, in such cases, the predictions of the forward model and the ideomotor account would not necessarily diverge.

### Quantification and statistical analysis

All analyses were conducted in R 4.3.1.^70^ For the analyses we only considered responses that were categorized as hits (excluding central hits in Experiment 1) or near misses. For the main analysis on the SoA we further only considered trials for which an SoA rating was available. Hits were considered to reflect an appropriate motor activity and misses to reflect an inappropriate motor activity. Hit feedback was considered to reflect intended feedback and miss feedback was considered to reflect unintended feedback. The visual feedback (intended vs. unintended) and motor activity (appropriate vs. inappropriate) variables were contrast coded^71^ using two separate orthogonal contrasts that reflected the pattern of the SoA as predicted by the classical comparator model and the ideomotor model (see Figure 3). Thus, for the four combinations of visual feedback and motor activity — that is, intended (visual feedback)/appropriate (motor activity), intended/inappropriate, unintended/appropriate, and unintended/inappropriate — we used the contrast (1 -1 -1 1) for the classical comparator model and the contrast (1 1 -1 -1) for the ideomotor model, with each element of these contrast vectors reflecting the weight for the respective combination of visual feedback and motor activity. For the classical comparator model, the contrast reflects the following assumption regarding the pattern of SoA ratings across variable combinations: intended/appropriate + unintended/inappropriate > intended/inappropriate + unintended/appropriate. For the ideomotor model, the contrast reflects the assumption: intended/appropriate + intended/inappropriate > unintended/appropriate + unintended/inappropriate (cf. Figure 3).

We then fit Bayesian mixed linear models^48^^,^^49^ separately for the classical comparator model and the ideomotor model, with the SoA rating as the dependent variable and the respective contrast for visual feedback and motor activity as the independent variable (fixed effect), as well as random person intercepts. This resulted in two Bayesian mixed linear models for Experiment 1, as there was only one near miss category. On average, the following number of trials was included in the analysis for Experiment 1: 29.25 trials (6% of the total number of trials, *SD* = 10.98) for which an appropriate motor activity resulted in intended visual feedback, 29.13 trials (6%, *SD* = 10.73) for which an appropriate motor activity resulted in unintended visual feedback, 46.63 trials (10%, *SD* = 11.23) for which an inappropriate motor activity resulted in intended visual feedback, and 45.00 trials (9%, *SD* = 0.70) for which an appropriate motor activity resulted in unintended visual feedback. In Experiment 2, however, there were several near miss categories depending on the distance of the response to the target area. Here we fit separate models for the different near miss categories (note that responses with actual hits, i.e., an appropriate motor activity resulting in intended or unintended feedback, were the same for all models). We fit separate models, because this allows to directly assess whether the classical comparator or the ideomotor model fit the data better when considering deviations from the target area of different magnitudes. In addition, it allowed us to specify concrete stopping criteria for our sequential sampling approach. For the main analysis, we considered the three near miss categories closest to the target area (i.e., within 40 px, between 40 and 80 px, and between 80 and 120 px from the target area). This covered most responses, as, on average, only 20% of responses fell into a more distant category. On average, the following number of trials was included in the analysis for Experiment 2: 17.38 trials (4%, *SD* = 8.39) for which an appropriate motor activity resulted in intended visual feedback, 18.25 trials (4%, *SD* = 8.91) for which an appropriate motor activity resulted in unintended visual feedback, 17.44 trials (4%, *SD* = 5.35, within 40 px), 15.94 trials (3%, *SD* = 5.47, between 40 and 80 px) trials, and 10.06 trials (2%, *SD* = 3.87, between 80 and 120 px) for which an inappropriate motor activity resulted in intended visual feedback, respectively, and 21.44 trials (4%, *SD* = 6.79, within 40 px), 13.81 trials (3%, *SD* = 5.47, between 40 and 80 px) trials, and 10.50 trials (2%, *SD* = 4.00, between 80 and 120 px) for which an inappropriate motor activity resulted in unintended visual feedback, respectively. For each contrast in the Bayesian mixed linear models we computed the mean of the posterior distribution (*M*) and the 95% highest posterior density interval (HDI). We then compared the models reflecting the prediction of the classical comparator model and the models reflecting the prediction of the ideomotor model via Bayesian model comparisons by computing Bayes factors (BF) in favor of the ideomotor model. In doing so, we test which of the assumptions mathematically expressed in the contrast coding (of the classical comparator model or the ideomotor model) fit the observed data better. A BF > 1 indicates evidence in favor of the ideomotor model and a BF < 1 indicates evidence in favor of the classical comparator model, whereby BF < 0.33 or > 3 are typically considered substantial evidence.^68^

In a post-hoc analysis for Experiment 2 we also tested for an effect of the distance of the response from the center of the target area on the SoA, considering all misses that were within 400 px to the target area (i.e., near misses). The distance was computed as the absolute difference between the endpoint of the mouse cursor and the coordinate of the center of the target area, considering only the x coordinate (for target areas at the top or bottom) or only the y coordinate (for target areas to the left or right). We then compared a model with the effect-coded visual feedback variable (1 = intended, -1 = unintended) with a model with an additional distance variable as a covariate and computed the BF in favor of an effect of distance. The models further included random person intercepts.

As a supplementary analysis, we investigated movement initiation times in the secondary task. We only considered trials for which initiation times were available and excluded trials with initiation times faster than 10 ms (indicating premature response onset) or slower than 3 s. This led to the exclusion of 31% of trials in Experiment 1 and 16% of trials in Experiment 2. We tested the influence of visual feedback and motor activity, as well as their interaction, on initiation times. Visual feedback and motor activity were effect coded (1 = intended or appropriate, -1 = unintended or inappropriate) and served as the independent variables (fixed effects). Models further included random person intercepts. We evaluated main effects and the interactions by conducting nested model comparisons by computing BF in favor of the respective effect. For evaluating main effects, a model with the respective independent variable was compared to an intercept-only model. For evaluating the interaction effect, the full model was compared to a model with both main effects but no interaction. Models were fit and Bayes factors were computed using the R package BayesFactor 0.9.12-4.6^72^ using the package’s default priors.

### Additional resources

Both experiments were preregistered prior to data collection (Experiment 1: https://doi.org/10.17605/OSF.IO/TJM6Q, Experiment 2: https://doi.org/10.17605/OSF.IO/FRM8U).
