# Supplementary material for: Goals rather than predictions determine the sense of agency
Source: iScience. 2025 May 5;28(6):112583. doi: 10.1016/j.isci.2025.112583 (PMC12159484; doi:10.1016/j.isci.2025.112583)

iScience, Volume *28*

Supplemental information

Goals rather than predictions determine the sense of agency

Marcel R. Schreiner, Bence Neszmélyi, Katharina A. Schwarz, and Wilfried Kunde

Figure S1. Kernel Density Plots Showing the Distribution of the Distance of the Participants' Movement Endpoints to the Center of the Target Area by Position of the Target Area for Experiments 1 and 2.

Trials in Which Participants Hit the Opposite Side of the Screen from the Target Area were Removed (a Total of 21 Trials in Experiment 1 and 14 Trials in Experiment 2). Vertical Dashed Lines Reflect the Borders of the Response Categories. For Experiment 1, These are Central Hits (Dark Green), Outer Hits (Light Green), and Near Misses (Yellow). For Experiment 2, these are Hits (Light Green) and Near Misses (Yellow), with the Borders of the Three Near Miss Categories Closest to the Target Area Being Displayed.

Experiment 1

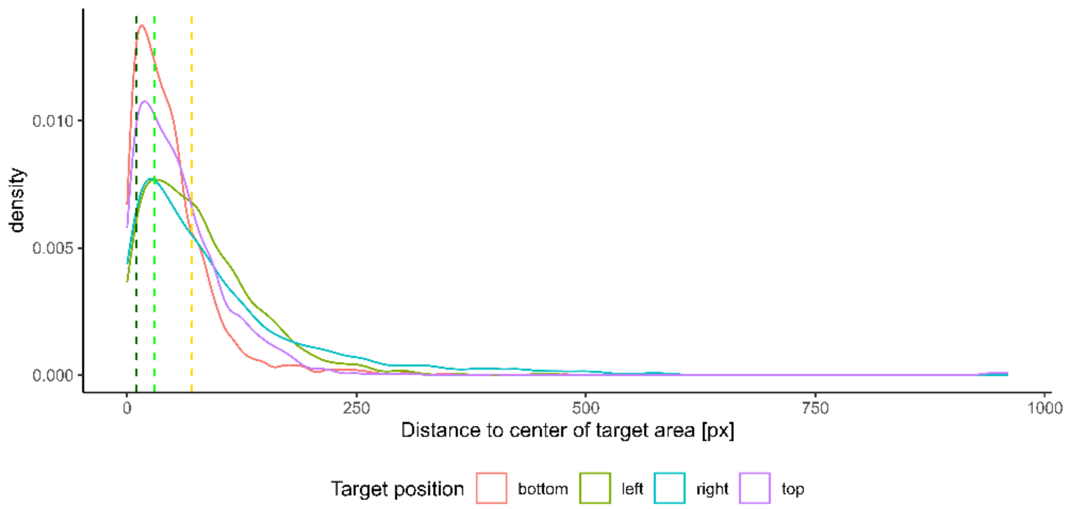

Experiment 2

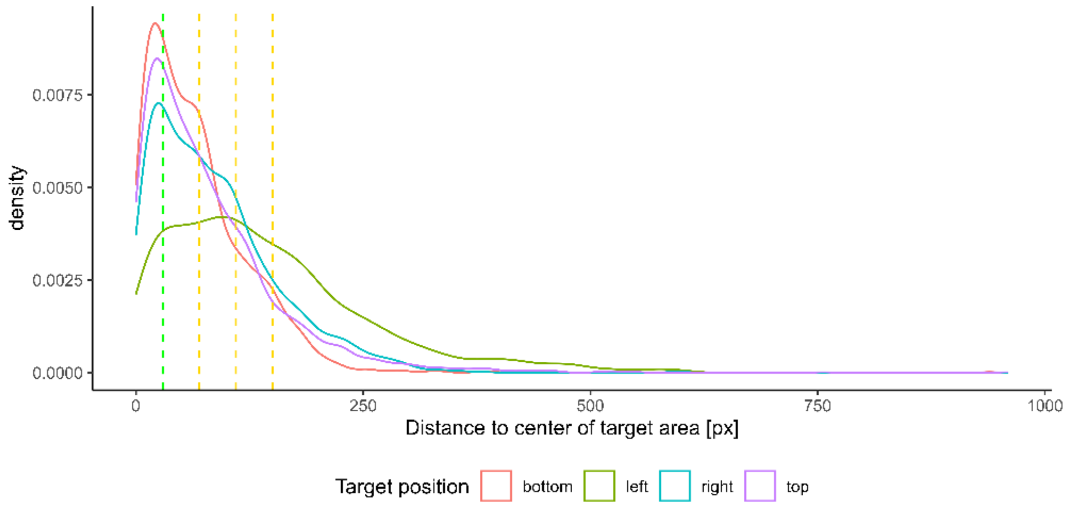

Supplement: Document S1. Figure S1 [file mmc1.pdf]
